# A Feature-Selective Independent Component Analysis Method for Functional MRI

**DOI:** 10.1155/2007/15635

**Published:** 2007-11-18

**Authors:** Yi-Ou Li, Tülay Adalı, Vince D. Calhoun

**Affiliations:** ^1^Department of Computer Science and Electrical Engineering, University of Maryland Baltimore County, 1000 Hilltop Circle, Baltimore, MD 21250, USA; ^2^The MIND Institute, University of New Mexico, Albuquerque, NM 87106, USA; ^3^Department of ECE, University of New Mexico, Albuquerque, NM 87106, USA; ^4^Department of Psychiatry, Yale University, New Haven, CT 06520, USA

## Abstract

In this work, we propose a simple and effective scheme to incorporate prior knowledge about the sources of interest (SOIs) in independent component analysis (ICA) and apply the method to estimate brain activations from functional magnetic resonance imaging (fMRI) data. We name the proposed method as feature-selective ICA since it incorporates the features in the sample space of the independent components during ICA estimation. The feature-selective scheme is achieved through a filtering operation in the source sample space followed by a projection onto the demixing vector space by a least squares projection in an iterative ICA process. We perform ICA estimation of artificial activations superimposed into a resting state fMRI dataset to show that the feature-selective scheme improves the detection of injected activation from the independent component estimated by ICA. We also compare the task-related sources estimated from true fMRI data by a feature-selective ICA algorithm versus an ICA algorithm and show evidence that the feature-selective scheme helps improve the estimation of the sources in both spatial activation patterns and the time courses.

## 1. INTRODUCTION

Independent component analysis is an exploratory data analysis technique to extract statistically independent sources from a given set of linear mixtures. Since the initial application of ICA to fMRI data [1], it has been demonstrated to be effective for studying brain function in numerous cases, (see e.g., [2, 3]). In the applications of ICA to fMRI data, the underlying sources are assumed to be either spatially independent activation patterns (spatial ICA) or independent temporal waveforms of blood oxygen level dependent (BOLD) signals (temporal ICA) [4]. In either case, the sources to be estimated from the fMRI data usually assume certain contextual features in their spatial or temporal domain. For example, due to the localization of brain function, the activation pattern of the physiologically interesting sources tend to be spatially smooth and clustered [5–7]. Similarly, the time courses of the physiologically interesting sources are more likely to assume a slow varying temporal pattern caused by the blood oxygen level dependent (BOLD) effect [8]. These contextual features encoded in the signal sample space, *that is, the spatial or temporal space in which the source signals are represented*, are not exploited in a standard ICA framework. When the contextual features of the sources are available a priori, it is desirable to incorporate this knowledge into the ICA procedure to achieve better estimation of the underlying fMRI sources of practical interest.

To address the sample space features in ICA or blind source separation (BSS), both principled and heuristic methods have been introduced. Choudrey and Roberts [9] introduce a one-dimensional hidden Markov source model and propose an integrated learning algorithm for the hidden Markov sources and the demixing network under a Bayesian formalism. Rowe [10] proposes a Bayesian BSS framework incorporating the spatial voxel dependence to infer the activation and the reference time course from fMRI data. Though Bayesian inference is a typical statistical approach to incorporate priors, the formulation of the posterior for ICA sources with sample space modeling is usually complicated due to the large sample size of the fMRI spatial maps. Therefore, simplifications and approximations are made to obtain practical solutions for Bayesian methods.

On the other hand, a number of non-Bayesian methods show improved results with relatively simple manipulations. Contextual ICA [11] solves the ICA problem with an autoregressive source modeling and uses interleaved updates to optimize the source model parameters and the demixing matrix to maximize the total likelihood. When the sources are two- or three-dimensional spatial images, autoregressive source processes become, if at all possible, overcomplicated to derive. Semiblind ICA [12] improves the time course estimation during spatial ICA by regulating the estimated time courses with the derived BOLD reference signal. In this case, the a priori feature is applied to the demixing vectors in the spatial ICA model.

Motivated by [12], in this work, we show how a regulation on the sample space features of the independent sources can be imposed in an iterative manner to improve the ICA estimation and we introduce an effective implementation of the scheme. In this case, since the demixing vectors are the explicit parameters being optimized to solve the ICA problem, the changes made in the source sample space have to be translated into the perturbation on the demixing vectors. Specifically, as the first step in this approach, a filter is designed to be selective to the sample space features of the source of interest (SOI). Within an ICA iteration, a feature-selective filtering operation is applied to a set of restored SOI estimates followed by a least-squares projection of the filtered source estimates from sample space to the space spanned by the observed data. The least-squares projection results in a set of new demixing vectors that optimally represent the filtered source estimates as linear combinations of the observed data by minimizing the sum of square projection error. We study the convergence properties of the ICA algorithm equipped with the proposed scheme to show that it converges in favor of sources whose features match the characteristics of the filter. We also compare the sources estimated by ICA and the proposed feature-selective ICA on both simulated and true fMRI data and show that improvement on the source estimation is achieved by incorporating the feature-selective scheme.

The selection of SOI estimates is important to the proposed feature-selective scheme because feature-selective scheme only improves convergence to the sources with the specified features, that is, the SOIs. Hence, the proposed scheme should be applied within a set of component estimates converging to the SOIs. The selection of SOI estimates in feature-selective ICA is discussed in detail within the context of fMRI source estimation.

The rest of the article is organized as follows Section 2 gives the feature-selective filtering-projection scheme and summarizes the sample space features of the fMRI sources. Section 3 shows the experimental results of the feature-selective ICA algorithm on simulated and true fMRI data. Discussions and concluding remarks are given in Sections 4 and 5, respectively.

## 2. METHOD

### 2.1. Feature-selective filtering-projection in ICA

The ICA generative model can be stated as x[n]=As[n], where s[n] is the vector of source components with the sample index n, A is a nonsingular mixing matrix, and x[n] is the vector of observed mixtures. We assume that the estimation is carried out in batch mode on all the available data. Therefore, we can drop the sample index n and expand each component as a row vector of samples arranged in its natural order, for example, as a time sequence or image contrast from ordered spatial locations. The ICA model thus becomes

(1) X=AS,

where X and S are matrices whose rows contain samples from each observed mixture and source component, respectively. The task of ICA is then to find a demixing matrix W such that the original components are recovered as

(2) $$\hat{\text{S}}=\text{WX}.$$

In an iterative ICA algorithm, the demixing matrix is updated recursively by the derived learning rule to achieve maximal independence among the source components. Within one iteration of such an algorithm, the feature-selective filtering-projection can be carried out by the following procedure:

- restoration: s^(k)=XTw(k), where k is the iteration index of the ICA algorithm;
- filtering: s^′(k)=Hs^(k), where filtering is expressed as premultiplication of the signal vector with the filtering matrix H defined by the feature-selective filter. The filter coefficients are normalized to have unit filtering gain;
- projection: w′(k)=(XXT)−1Xs^′(k), that is, the least-squares solution, min⁡w∥s^′(k)−XTw∥2.

We expand ${\text{w}}^{\prime }(k)$ in (iii) as

(3) $${\text{w}}^{\prime }(k)=(\text{X}{\text{X}}^{T}{)}^{-1}\text{A}\text{S}\text{H}{\text{S}}^{\text{T}}{\text{A}}^{T}\text{w}(k),$$

where (1) and the expressions in step (i) and (ii) are used.

We define $\Sigma_{ss^{\prime }}≜(1/N)\text{S}\text{H}{\text{S}}^{T}=(1/N)\text{S}\text{S}{}^{\prime T}$ as the feature-selective correlation matrix of the original sources and the filtered ones where N is the total number of samples.

Since ${\text{S}}^{\prime }$ is the filtered counterpart of the independent components S, $\Sigma_{ss^{\prime }}$ is approximately a diagonal matrix with the correlation coefficients $r_{s_{i}s_{i^{\prime }}}=(1/N){\text{s}}_{i}^{T}s_{i^{\prime }},i=1,2,...,$ on its main diagonal. By substituting $\Sigma_{ss^{\prime }}$ into (3), we obtain

(4) $${\text{w}}^{\prime }(k)={\left(\frac{1}{N}\text{X}{\text{X}}^{T}\right)}^{-1}\text{A}\Sigma_{ss^{\prime }}{\text{A}}^{T}\text{w}(k).$$

This equation reveals the effect of the feature-selective filtering-projection operation on the demixing vector and leads to the following lemma.

**Lemma 1**

For a whitened mixture of independent sources, if an iterative ICA algorithm converges to a demixing vector, ${\text{w}}_{i}$, corresponding to one SOI, $s_{i}$, the incorporation of the feature-selective filtering-projection will increase the convergence rate of each element in ${\text{w}}_{i}$, to the true demixing vector, by a factor $\left|r_{s_{i}s_{i^{\prime }}}/r_{s_{j}s_{j^{\prime }}}\right|$, ∀j≠i.

The Lemma shows an observed property of the feature-selective scheme, that is, the iterative feature-selective ICA algorithm is locally stable and has faster convergence for the SOIs. We give the proof of the Lemma in the Appendix.

Therefore, if the designed feature-selective filter is selective to the desired sample space features such that the filtered fMRI source signal $s_{i^{\prime }}$ is highly correlated with the original signal compared to the non-SOIs, that is, $|r_{s_{i}s_{i^{\prime }}}/r_{s_{j}s_{j^{\prime }}}|>1\;\forall j\neq i$, the incorporation of the feature-selective projection will accelerate the convergence of the corresponding demixing vector ${\text{w}}_{i}$ to the true demixing vector.

### 2.2. Features of fMRI data in the sample spaces

FMRI data can be decomposed into either a set of independent spatial activation patterns with the corresponding time courses using spatial ICA, or a set of independent BOLD time sequences with the related spatial distribution maps using temporal ICA. In either case, the underlying SOIs possess certain features in the sample space.

In spatial domain, some activation regions can be hypothesized through the study of, for example, the resting state fMRI data [13–15]. These hypothesized activation regions can be incorporated into ICA as the location features of potential SOIs. Furthermore, SOIs that are task-related, transiently task-related, and function-related tend to assume smooth and localized (clustered) characteristics on the activation maps.

In temporal domain, task-related time courses assume varying pattern similar to the task paradigm, hence, the paradigm information can be incorporated into ICA to improve the estimation of task-related sources [12]. Moreover, all functional time courses assume low frequency temporal variations due to the modulation by the hemodynamic response function [16, 17]. The physiology-related time courses, such as the heart beat and breathing, tend to have low frequency spectra [18].

On the other hand, for some fMRI artifacts, for example, motion-related sources, the spatial maps assume high-spatial frequency patterns such as edges and the time courses assume high-pass temporal patterns such as abrupt changes.

All these features can be enhanced or attenuated by the spatial or temporal filters with properly selected parameters. These filters are, thus, used in the feature-selective scheme to regulate the ICA estimation.

## 3. EXPERIMENT

We use the Infomax algorithm [19] incorporated with the proposed feature-selective scheme to perform the feature-selective ICA (FS-ICA) and compare the SOIs estimated by FS-ICA with those by ICA. We perform experiments on resting state fMRI data with superimposed activations as well as fMRI data from a visuomotor task.

### 3.1. ICA estimation on hybrid fMRI data

**Figure 1:**
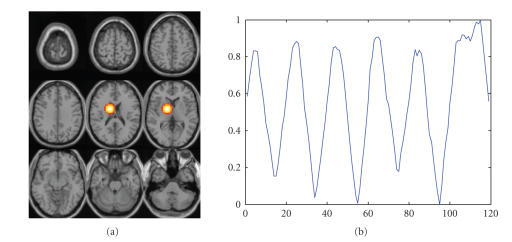
Simulated source superimposed on a resting state fMRI dataset: (a) activation region, (b) time course.

To generate the hybrid fMRI data, a 30 × 30 × 25 mm^3^ activation region with irregular shape is created at a chosen anatomical location in the brain, together with a time course simulating the hemodynamic response to a box-car task paradigm. The imposed activation region and the time course are shown in Figure 1.

The activation is weighted by the time course at each time point, forming a spatiotemporal activation pattern. This activation pattern is scaled with a factor, η, which is controlled by a prespecified contrast to noise ratio (CNR) defined as

(5) $$\text{C}\text{N}\text{R}=\frac{\Delta |S{|}_{\text{m}\text{a}\text{x}}}{\sigma_{t}},$$

where $\Delta |S{|}_{\text{m}\text{a}\text{x}}$ is the maximum activation amplitude, $\sigma_{t}$ is the square root of the noise variance at the activated area. To generate the simulated dataset, CNR is set to 1, which is a typical value for a robust task paradigm. The scaled spatiotemporal activation pattern is then added to a resting-state fMRI dataset to form a hybrid fMRI dataset.

The data is dimension reduced to 15 principal components using principal component analysis and ICA is performed on the preserved principal components.

To test the effect of incorporating prior knowledge about the activation to the ICA estimation performance, we generate a series of a priori activation templates overlapping with the imposed activation at different degrees ranging from 100% down to 10%. During the iterative ICA estimation, the spatial sources are restored and the SOI is selected by spatially correlating the current source estimate with the a priori activation template. Feature-selective filtering is then performed on the selected SOI estimate by masking the SOI estimate using the template. This filtering is equivalent to an all-pass spatial filter at the a priori activation region and an all-stop filter elsewhere. The new demixing vector is obtained as the least-squares projection of the filtered SOI estimate onto the space spanned by the whitened mixtures, that is, as step (iii) in Section 2.1.

After ICA, the SOI is selected by the activation template and thresholded based on Z-score, that is, the normalized activation levels within each map, to mark out the activated voxels. The use of Z-score is equivalent to a normalization of the map to resolve the scaling ambiguity inherent in ICA. Different Z-score thresholds lead to differences in regions identified as activated areas. The detected regions are compared with the true artificial activation template to calculate the true positive rate (TPR) and the false positive rate (FPR). The TPR is calculated by

(6) $$\frac{\text{V}\text{O}{\text{X}}_{\text{P}\text{O}\text{S}⋂\text{A}\text{C}\text{T}}}{\text{V}\text{O}{\text{X}}_{\text{A}\text{C}\text{T}}},$$

where $\text{V}\text{O}{\text{X}}_{\text{P}\text{O}\text{S}⋂\text{A}\text{C}\text{T}}$ is the number of positive voxels overlapping with the activated voxels on the template and $\text{V}\text{O}{\text{X}}_{\text{A}\text{C}\text{T}}$ is the total number of activated voxels on the template. The FPR is calculated as

(7) $$\frac{\text{V}\text{O}{\text{X}}_{\text{P}\text{O}\text{S}⋂\text{n}\text{o}\text{A}\text{C}\text{T}}}{\text{V}\text{O}{\text{X}}_{\text{n}\text{o}\text{A}\text{C}\text{T}}},$$

where $\text{V}\text{O}{\text{X}}_{\text{P}\text{O}\text{S}⋂\text{n}\text{o}\text{A}\text{C}\text{T}}$ is the number of positive voxels overlapped with the nonactivated voxels on template and $\text{V}\text{O}{\text{X}}_{\text{n}\text{o}\text{A}\text{C}\text{T}}$ is the total number of nonactivated voxels on the template. A receiver operating characteristic (ROC) plot is then formulated based on the TPR and FPR at different thresholds.

**Figure 2:**
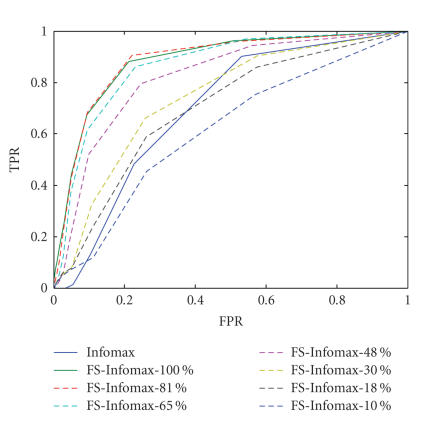
ROC plots for detection of the superimposed activation in the hybrid fMRI data with FS-Infomax incorporating a priori activation templates overlapping with the true activation region to different degrees (100%–10%), and ROC plot for detection of the superimposed activation with Infomax estimation.

Figure 2 shows the ROC curves for detection of the imposed activation using FS-Infomax with a priori activation templates overlapping with the true activation to different degrees. It is observed that the Infomax algorithm with feature-selective scheme improves the estimation of the superimposed activation by incorporating a priori location information about the activation. The degree of improvement decreases as the prior deviates from the ground truth. When the a priori template poorly matches the true activation, that is, overlaps at 10%, the detection performance is decreased to the same level as the Infomax algorithm. It is worth noting that for the range of FPR∈[0,0.1], which is usually of more practical interest for detection performance, FS-Infomax with all a priori templates show significant improvement on detection power compared to Infomax. The results indicate that the feature-selective scheme is effective to the detection of activation at relatively low CNR conditions.

### 3.2. Spatial ICA of fMRI data from a visuomotor paradigm

#### 3.2.1. Participants and experimental paradigm

Twelve right-handed participants with normal vision—six females, six males, average age 30 years—participated in the study. Subjects performed a visuomotor task involving two identical but spatially offset, periodic, visual stimuli, shifted by 20 seconds from one another. The visual stimuli were projected via an LCD projector onto a rearprojection screen subtending approximately 25 degrees of visual field, visible via a mirror attached to the MRI head coil. The stimuli consisted of an 8 Hz reversing checkerboard pattern presented for 15 seconds in the right visual hemifield, followed by 5 seconds of an asterisk fixation, followed by 15 seconds of checkerboard presented to the left visual hemifield, followed by 20 seconds of asterisk fixation. The 55-second set of events was repeated four times for a total of 220 seconds. The motor stimuli consisted of participants touching their thumb to each of their four fingers sequentially, back and forth, at a self-paced rate using the hand on the same side on which the visual stimulus is presented.

#### 3.2.2. Imaging parameters

Scans were acquired at the Olin Neuropsychiatry Research Center at the Institute of the Living on a Siemens Allegra 3T dedicated head scanner equipped with a 40mT/m gradients and a standard quadrature head coil. The functional scans were acquired using gradient-echo echo planar imaging with the following parameters: repeat time (TR) = 1.50 seconds, echo time (TE) = 27 milliseconds, field of view = 24 cm, acquisition matrix = 64 × 64, flip angle = 60 degrees, slice thickness = 4 mm, gap = 1 mm, 28 slices, ascending acquisition. Six “dummy” scans were performed at the beginning to allow for longitudinal equilibrium, after which the paradigm was automatically triggered to start by the scanner.

#### 3.2.3. Data analysis

**Preprocessing**

Data were processed using the MATLAB Toolbox for Statistical Parametric Mapping (SPM).^1^ Images were realigned using INRIAlign—a motion correction algorithm unbiased by the local signal changes [20, 21]. Data were spatially normalized into the standard Montreal Neurological Institute space [22]. The data (originally acquired at 3.75 × 3.75 × 5 mm^3^) were slightly resampled to 3 × 3 × 5 mm^3^, resulting in 53 × 63 × 28 voxels. The data is spatially smoothed with an 8 × 8 × 8 mm^3^ FWHM Gaussian kernel, resulting in the smoothed fMRI dataset.

**Denoising by signal and noise subspace decomposition**

Due to the relatively low CNR of the fMRI data, it is reasonable to incorporate a noise model into the data analysis. Here, we adopt a signal-noise subspace decomposition by principal component analysis (PCA) where the dimension of the signal subspace, that is, the number of informative principal components, is selected based on an improved implementation of the information-theoretic criteria [23]. A noiseless ICA is thus performed within the signal subspace to estimate an equal number of independent components to the number of the preserved principal components.

**Feature-selective scheme**

To impose the spatial smoothness on the sources, we adopt a 3 × 3-voxel two-dimensional neighborhood system, that is, a 3 × 3-voxel clique [24], and convolve each slice of the current source estimate with the normalized 3 × 3 smoothing kernel, that is, all the coefficients of the kernel are equal and sum up to unity. This is equivalent to a 9 × 9 mm^2^ spatial span based on the voxel size of the fMRI data. To avoid oversmoothing of the activation areas, the smoothing is applied adaptively on the areas below a prespecified z-score threshold ($Z_{\text{t}\text{h}}=1$), that is, voxels with magnitude greater than one standard deviation of the voxel value distribution within the activation map. In this experiment, we apply the feature-selective scheme to two task-related SOIs, which are introduced in previous fMRI studies for a similar visuomotor paradigm [25]. To identify these SOIs, we use the activation templates generated in [26]. The corresponding Brodmann areas (BAs) are BAs 1, 2, 3; BA 4; BA 6; BA 17; BAs 18, 19 and the right and left hemispheres containing the above regions are chosen to form the right and left task-related templates, respectively. The SOIs are identified by correlating the source estimates at each iteration with the templates.

**ICA estimation**

Group ICA [3] is performed on the twelve subjects data using Group ICA of fMRI Toolbox (GIFT).^2^

**Figure 3:**
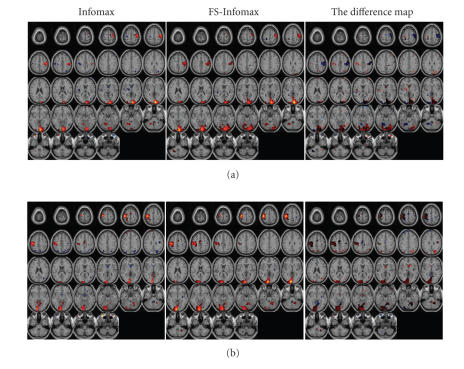
Activation maps of the two task-related components using group t-statistics from the group ICA results. *Left*: Infomax, *center *: FS-Infomax, *right*: the difference map (FS-Infomax-Infomax): (a) right visuomotor activation, (b) left visuomotor activation.

The group activation maps shown on the left and center columns of Figure 3 are obtained by a two-tailed t-test, performed voxelwise upon the back-reconstructed activation maps of twelve subjects for each SOI, which corresponds to a random effect inference [3]. The presented group activation maps for Infomax (Figures 3: left column) and Feature Selective Infomax (Figure 3: center column) are thresholded based on the t-statistics corresponding to significance level α=0.05, for sample, size N=12. The super-thresholded voxels are color-coded according to the mean activation level of twelve subjects and overlaid onto the brain anatomical template. For comparison, the right column of Figure 3 shows the difference maps (FS-Infomax-Infomax) of each SOI, obtained by taking the difference of the mean activation levels at locations that are tested to be activation for *either* Infomax *or* FS-Infomax.

It is observed that (i) based on the same statistical threshold, larger activation regions are identified in the case of FS-Infomax compared to Infomax; (ii) the group activation maps obtained by FS-Infomax assume higher mean activation level on the primary visual cortex; (iii) there is a decrease for the activation level on the primary motor cortex, meanwhile, the negative activation on the opposite side motor cortex decreases to below the threshold level.

**Figure 4:**
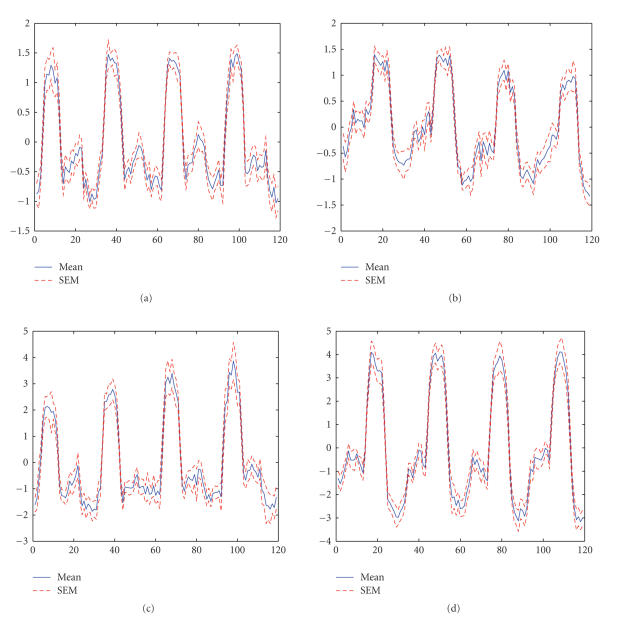
Averaged time courses of the two task-related components with the standard error of the mean (SEM). (a) Right visuomotor time course of Infomax, (b) left visuomotor time course of Infomax, (c) right visuomotor time course of FS-Infomax, (d) left visuomotor time course of FS-Infomax.

Furthermore, correlation between the estimated time courses and the corresponding task paradigm is calculated and averaged among the twelve subjects. The task-related time courses estimated by FS-Infomax assume more significant correlation with the task paradigm (0.75±0.11 for the right visuomotor task and 0.80±0.06 for the left visuomotor task), compared to the time courses estimated by Infomax algorithm (0.66±0.18 for the right visuomotor task and 0.62±0.13 for the left visuomotor task). The mean time courses are shown in Figure 4 together with the standard error of the mean (SEM). This indicates that the activation regions estimated for the two task-related components by FS-Infomax are more significantly related to the visuomotor task performed.

### 3.3. Temporal ICA of fMRI data

Temporal ICA [2,27,28] can be performed to estimate the independent time courses from the fMRI data. When temporal ICA is applied to the fMRI data, the spatial voxels correspond to the dimension of the input and the time points correspond to the samples of signals.

**Figure 5:**
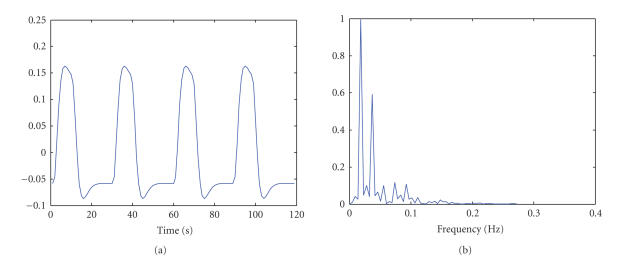
The temporal waveform and frequency spectrum of the predicted right visuomotor time course: (a) temporal waveform, (b) frequency spectrum.

We select the two task-related time courses as the SOIs and generate the predicted task-related time courses by convolving the task paradigm (box-car pattern) with the hemodynamic response function. As an example, the predicted right visuomotor task-related time course and its frequency spectrum are shown in Figure 5. It is observed that the major spectral power is concentrated in the frequency band from 0.01 to 0.1 Hz. Hence, a temporal FIR filter with a relaxed passband of 0.01 to 0.3 Hz is designed as the feature-selective filter for the two task-related SOIs.

Since the expected task-related time courses have subGaussian distribution on the sampled values, extended Infomax algorithm is used [29]. We apply temporal ICA to the fMRI data of one subject from the group performing the same task as described in the previous section. We pick one fMRI slice cutting through the visual cortex to perform temporal ICA. During temporal ICA estimation, two SOIs are selected by correlating source estimates with the predicted task-related time courses. The demixing vectors are obtained by least-squares projection of the lowpass filtered time course estimates onto space spanned by the whitened mixtures, that is, as step (iii) in Section 2.1.

**Figure 6:**
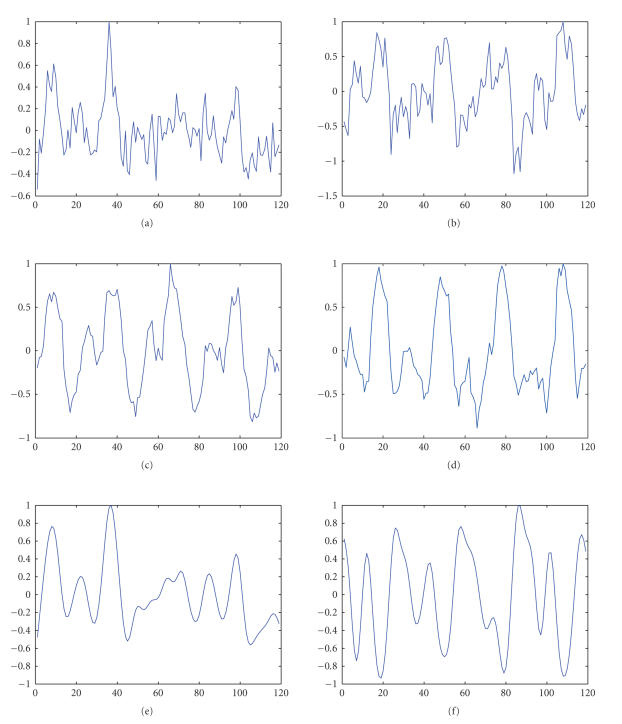
Results of temporal ICA: (a) right visuomotor time course estimated by extended Infomax, (b) left visuomotor time course estimated by extended Infomax, (c) right visuomotor time course estimated by FS-Infomax, (d) left visuomotor time course estimated by FS-Infomax, (e) right visuomotor time course estimated by extended Infomax with post feature-selective filtering, (f) left visuomotor time course estimated by extended Infomax with post feature-selective filtering; $r_{tt_{0}}$ is the calculated correlation with the corresponding task paradigm: (a) $r_{tt_{0}}=0.69\pm 0.01$, (b) $r_{tt_{0}}=0.66\pm 0.01$, (c) $r_{tt_{0}}=0.79\pm 0.01$, (d) $r_{tt_{0}}=0.87\pm 0.01$, (e) $r_{tt_{0}}=0.76\pm 0.01$, (f) $r_{tt_{0}}=0.70\pm 0.01$.

Twenty Monte Carlo trials are performed in each case of (1) extended Infomax, (2) FS-Infomax, and (3) extended Infomax with the estimated time courses filtered by the feature-selective filter after ICA estimation, that is, a post feature-selective filtering. For each trial, ICA algorithm processes the data blocks with random order.^3^ Figure 6 shows the two estimated task-related time courses in the three cases and correlations of the time courses with the corresponding task paradigm. It is observed that the task-related time courses estimated by FS-Infomax appear less noisy and achieve the highest correlation with the task paradigm among the three cases. Althoughpost feature-selective filtering seemingly increases correlation with the task-paradigm, it causes loss of phase information as observed in Figures 6(e) and 6(f).

## 4. DISCUSSION

### 4.1. The effect of feature-selective scheme on fMRI source estimation

In this work, we focus on the smoothness of spatial activation maps and the lowpass features of task-related time courses, estimated as the independent sources in ICA of fMRI data, though, a number of sample space features can be utilized—as discussed in Section 2.2. The experimental results in Section 3.2 show improved estimation on the activation maps by FS-ICA in that (i) larger brain regions that are expected to be activated by the performed task are identified; and (ii) for the task-related components, the corresponding time courses assume higher correlation with the task-paradigm, suggesting that the increased activation areas estimated by FS-ICA consist of task-related voxels not identified by ICA.

In Section 3.2, we performed group ICA instead of single subject ICA to increase the statistical power of estimation. Hence, the SOIs obtained by group ICA are common activations across subjects. There are, however, activations as well as artifacts specific to individual subjects. The subject specific characteristics can be studied by comparing the single subject ICA results with the group ICA results, which is a topic of its own interest. Our feature-selective scheme imposing spatial smoothness can also be applied to single subject spatial ICA based on the features of brain activation patterns discussed in Section 2.2. We performed similar comparisons between ICA and FS-ICA on each of the twelve single subjects and observed similar improvement on the SOIs with slightly decreased consistency.

We include an example of temporal ICA in Section 3.3 and show that the proposed feature-selective scheme can be implemented in a different sample space, the temporal domain, to improve the estimation of physiologically interesting time courses. In temporal ICA of fMRI data, singular value decomposition can be applied to achieve dimension reduction by principal component projection, hence, avoiding calculation of sample covariance matrix with the large spatial dimension of the fMRI data. On the other hand, a single slice containing the brain regions of interest, or a specific brain region of interest, is favorable for the practice of temporal ICA [2, 27, 28]. The utility and implementation of temporal ICA for fMRI data is, though, an interesting topic for further investigation.

It is worth noting that the feature-selective scheme we introduce, which imposes spatial smoothness, is different from the use of spatial smoothing as a preprocessing step in fMRI data analysis. Feature-selective scheme imposes a smoothness constraint on the underlying sources of interest through the corresponding demixing vectors. Smoothing as a preprocessing step is used to suppress high frequency noise in the fMRI data universally and to minimize the impact of spatial variability among subjects. Therefore, design considerations on the smoothing kernel are different for the two cases. The smoothing filter in FS-ICA is specified through prior knowledge on the location and smoothness of potential SOIs, such that it can impose an impact significant enough for altering the demixing vectors through a least-squares projection. The smoothing kernel used in the preprocessing step, however, is usually a Gaussian function with a size comparable to the voxel size of fMRI data and should be chosen carefully to avoid oversmoothing and hence loss of relevant information. Moreover, a feature-selective scheme with spatial smoothing can improve the estimation of sources from fMRI data that are not presmoothed. We perform the same experiments on unsmoothed fMRI data to compare the estimated SOIs by ICA and feature-selective ICA and observe similar improvement on the activation regions (results not shown). Since presmoothing is a typical step in current fMRI analysis given the relatively low CNR of the fMRI acquisition, we focus on the application of the method to the presmoothed data.

The partial smoothing we implement in the experiments pushes the background of activation map toward a more Gaussian distribution while leaving the activated regions at the tails of the distribution unchanged. The overall distribution, however, keeps to be super Gaussian, which is the favorable source distribution for ICA [19, 30].

### 4.2. The implementation of feature-selective scheme

Since a specific feature-selective filter only enhances estimation of the corresponding SOI(s), the feature-selective scheme is essentially a per-component implementation in ICA estimation. Therefore, a strategy for selecting the potential SOI estimates is necessary before the application of feature-selective scheme. For fMRI data analysis, the potential SOIs can be picked out by different measures, such as spatial sparsity, degree of BOLD modulation in temporal domain, skewness of the voxel value distribution, or a combination of such [31]. Classification of the component estimates into SOIs and non-SOIs can be achieved with these measures using a pattern recognition technique such as discriminant analysis. Usually though, expert knowledge is required to train the classification network. On the other hand, a priori activation templates can be obtained by, for example, the analysis of the resting-state fMRI data [13–15] and used for the selection of potential SOIs in a feature-selective ICA on fMRI data.

The selection of potential SOI estimates and hence the application of the feature-selective scheme should start after a preliminary convergence of the ICA algorithm when each component estimate starts assuming different sample space features. The preliminary convergence can be identified in the same way as the ICA iteration is terminated, with a more relaxed threshold on the change of the demixing vectors being optimized.

In the proposed scheme, the feature-selective filtering operation is represented by a matrix H such that, by multiplying the signal vector with matrix H, the signal is processed by the filtering sequences in each row of H. To address a global feature, H is formed by arranging one set of filtering sequence in a convolutive pattern in each row. In this case, the signal is passed through a linear shift-invariant system, as in the experiment of Section 3.3. For localized features, H may contain filtering sequences of different types of characteristics. This is equivalent to processing the signal with a linear shift-variant system, as in the experiments of Sections 3.1 and 3.2. The variation of the filter characteristics in H matrix, for example, defined by the a priori template, enables adaptation of different features at different locations in the source sample space.

### 4.3. The difference of feature-selective filtering-projection from pre- or postfiltering in ICA

The proposed feature-selective filtering-projection scheme in the ICA framework achieves a different objective compared to that of the filtering as a preprocessing step of ICA [32, 33].

In ICA, prefiltering is applied to the mixtures to remove unwanted signals such as out-of-band noise to enhance signal-to-noise ratio. Once the data are prefiltered, only the filtered part is used in the subsequent analysis, which might imply loss of relevant information from the original data.

Similarly, post filtering on the estimated components can not improve fMRI source estimation due to the loss of information, as observed in Figures 6(e) and 6(f).

ICA with iterative feature-selective filtering-projection scheme, on the other hand, keeps the original data as a mixture throughout the ICA process and applies the filtering selectively only for the SOIs. Hence, the overall effect of the scheme is to make the ICA algorithm converge in favor of the SOIs. Therefore, the proposed scheme works with a lossless data representation as most of the ICA algorithms.

### 4.4. Toward the framework of dynamic source model and Bayesian inference

Statistical models of fMRI spatial maps and time courses, and the associated inference methods such as variational Bayesian, are proposed for fMRI analysis by linear regression. Penny et al. [34] incorporate fMRI spatial dependence into the general linear model by introducing a Laplacian operator onto the regression coefficients and performing a variational Bayes inference. Woolrich et al. [35] propose a more involved spatiotemporal model on fMRI time series including autoregressive noise process and Markov random field spatial sources and utilize Markov Chain Monte Carlo to perform inference. Using Bayesian estimation to incorporate dynamic source priors into ICA/BSS is a promising direction in the field. Some of the relevant work are mentioned in the introduction of this article (see Section 1).

## 5. CONCLUDING REMARK

In this work, we propose a method to incorporate a priori knowledge about the source signals into ICA estimation by a feature-selective filtering-projection scheme. The method uses controlled feature-selective filtering on the iterative source estimates and projection based on the optimal estimators under the linear model. The feature-selective filtering is defined in a general sample space such as spatial domain for image processing or temporal domain in time sequence analysis. Compared to the classical Bayesian framework that incorporates statistical priors in the estimation, our approach uses linear filtering and linear space projection that is easier to implement and more readily accessible in signal processing applications. We demonstrate that the proposed scheme improves the estimation of spatial and temporal activation patterns in fMRI data, when incorporated into ICA algorithms.

## Footnotes

^1^ http://www.fil.ion.ucl.ac.uk/spm/software/spm2.

^2^ http://icatb.sourceforge.net.

^3^ http://www.sccn.ucsd.edu/eeglab/allfunctions/runica.html.

## APPENDIX

Proof Since A is assumed to be a nonsingular matrix, we can premultiply both sides of (4) with ${\text{A}}^{-1}$ to obtain

(A.1) $$A^{-1}{\text{w}}^{\prime }(k)=A^{-1}{\left(\frac{1}{N}XX^{T}\right)}^{-1}A\Sigma_{ss^{\prime }}A^{T}w(k).$$

Because the mixtures are prewhitened, we have

(A.2) $$\frac{1}{N}\text{X}{\text{X}}^{T}=\frac{1}{N}\text{A}\text{S}{\text{S}}^{T}{\text{A}}^{T}=\text{A}\Sigma_{s}{\text{A}}^{T}=\text{I},$$

where I is the identity matrix and $\Sigma_{s}≜(1/N)\text{S}{\text{S}}^{T}$ is the sample correlation matrix of the original source components. From the last equality in (A.2), we have

(A.3) $${\text{A}}^{T}=\Sigma_{s}^{-1}{\text{A}}^{-1}.$$

Substituting (A.3) and $(1/N)\text{X}{\text{X}}^{T}$ = I into (A.1), we obtain

(A.4) $${\text{A}}^{-1}w^{\prime }(k)={\overset{¯}{\Sigma }}_{ss^{\prime }}{\text{A}}^{-1}\text{w}(k),$$

where ${\bar{\Sigma }}_{ss^{\prime }}≜\Sigma_{ss^{\prime }}\Sigma_{s}^{-1}$. By the independence and equal-variance assumption, $\Sigma_{s}$ is a diagonal matrix with identical elements $\sigma_{s}^{2}=(1/N){\text{s}}_{i}^{T}s_{i}$ on its main diagonal. Since $\Sigma_{ss^{\prime }}$ is also diagonal, ${\bar{\Sigma }}_{ss^{\prime }}$ is a diagonal matrix with the normalized feature-selective correlation coefficients $r_{s_{i}s_{i^{\prime }}}/\sigma_{s}^{2}$ on its main diagonal. Now we define the *global* demixing vector $\text{z}(k)≜{\text{A}}^{-1}\text{w}(k)$ and rewrite (A.4) as

(A.5) $$z^{\prime }(k)={\overset{¯}{\Sigma }}_{ss^{\prime }}\text{z}(k).$$

Hence, the feature-selective filtering-projection is equivalent to premultiplication of z(k) with the normalized feature-selective correlation matrix ${\bar{\Sigma }}_{ss^{\prime }}$ within each iteration of the ICA algorithm. For ICA algorithms whose local convergence property has been shown, such as for Infomax [36], and FastICA [37], each global demixing vector z converges to a unit vector with only one nonzero element. Suppose we estimate the component $s_{i}$ whose global demixing vector is ${\text{z}}_{i}=[z_{1i},\ldots ,z_{ii},\ldots ,z_{mi}{]}^{T}$ converging to [0,…,c,…,0] where c is a nonzero quantity, when $s_{i}$ assumes the specified feature, we have $|r_{s_{j}s_{j^{\prime }}}/r_{s_{i}s_{i^{\prime }}}|<1,\forall j\neq i$. Hence, (A.5) becomes

(A.6) $$\begin{array}{ll} z_{i^{\prime }}(k) & =\frac{1}{\sigma_{s}}\text{diag}[r_{s_{1}s_{1^{\prime }}},\ldots ,r_{s_{i}s_{i^{\prime }}},\ldots ,r_{s_{m}s_{m^{\prime }}}]z_{i}(k)\\ & =\frac{r_{s_{i}s_{i^{\prime }}}}{\sigma_{s}}{\left[\frac{r_{s_{1}s_{1^{\prime }}}}{r_{s_{i}s_{i^{\prime }}}}z_{1i},\ldots ,z_{ii},\ldots ,\frac{r_{s_{m}s_{m^{\prime }}}}{r_{s_{i}s_{i^{\prime }}}}z_{mi}\right]}^{T}, \end{array}$$

which indicates that the decrease of $z_{ji}$, j≠i, to zero is accelerated by the factor $\left|r_{s_{j}s_{j^{\prime }}}/r_{s_{i}s_{i^{\prime }}}\right|$.
